# Structural Model for Transient Pt Oxidation during Fuel Cell Start-up Using Electrochemical X-ray Photoelectron Spectroscopy

**DOI:** 10.1021/acsami.2c09249

**Published:** 2022-07-29

**Authors:** Hassan Javed, Axel Knop-Gericke, Rik V. Mom

**Affiliations:** † Leiden Institute of Chemistry, Leiden University, PO Box 9502, Leiden 2300 RA, The Netherlands; ‡ Fritz Haber Institute of the Max Planck Society, Faradayweg 4-6, Berlin 14195, Germany; § Max-Planck-Institute for Chemical Energy Conversion, Stiftstrasse 34-36, Mülheim an der Ruhr 45413, Germany

**Keywords:** platinum oxidation, X-ray absorption spectroscopy, X-ray photoelectron spectroscopy, nanoparticles, electrocatalyst, graphene, Nafion, fuel cells

## Abstract

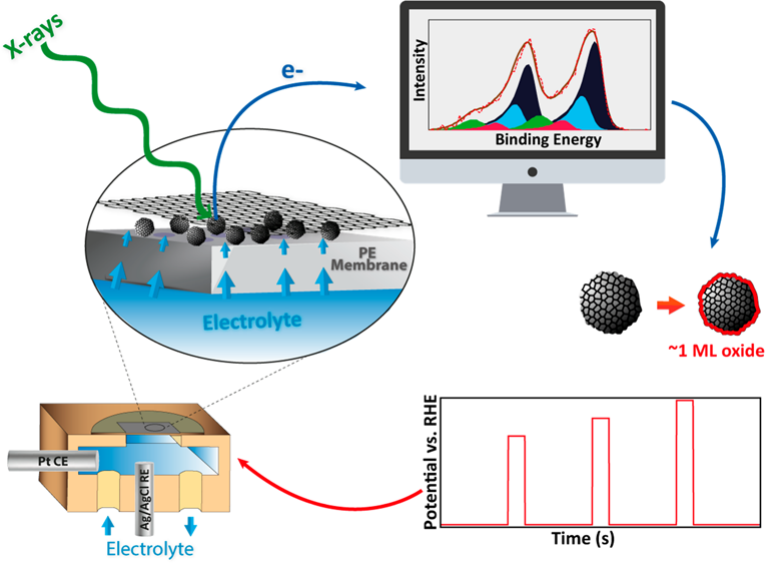

Potential spikes during the start-up and shutdown of fuel cells are a major cause of platinum electrocatalyst degradation, which limits the lifetime of the device. The electrochemical oxidation of platinum (Pt) that occurs on the cathode during the potential spikes plays a key role in this degradation process. However, the composition of the oxide species formed as well as their role in catalyst dissolution remains unclear. In this study, we employ a special arrangement of XPS (X-ray photoelectron spectroscopy), in which the platinum electrocatalyst is covered by a graphene spectroscopy window, making the in situ examination of the oxidation/reduction reaction under wet conditions possible. We use this assembly to investigate the change in the oxidation states of Pt within the potential window relevant to fuel cell operation. We show that above 1.1 V_RHE_ (potential vs reversible hydrogen electrode), a mixed Pt^δ+^/Pt^2+^/Pt^4+^ surface oxide is formed, with an average oxidation state that gradually increases as the potential is increased. By comparing a model based on the XPS data to the oxidation charge measured during potential spikes, we show that our description of Pt oxidation is also valid during the transient conditions of fuel cell start-up and shutdown. This is due to the rapid Pt oxidation kinetics during the pulses. As a result of the irreversibility of Pt oxidation, some remnants of oxidized Pt remain at typical fuel cell operating potentials after a pulse.

## Introduction

Fuel cells have the potential to play a pivotal role in the transition to sustainable energy systems. They are employed in a wide range of applications, including transportation, power generation, and material handling, offering a potentially CO_2_-free alternative to the traditional internal combustion engines.^1−4^ Presently, platinum is the best catalyst for polymer electrolyte fuel cells (PEMFCs), for both the anodic and the cathodic half reactions. However, the activity of the cathode decreases over time due to Pt dissolution, which causes a reduction in the electrochemically active surface area (ECSA).^5−7^ At present, this degradation of the cathode is limiting the lifetime of fuel cells.^4,5,8−10^ Hence, mitigating Pt dissolution is a key step in fuel cell development.

The degradation of fuel cell cathodes primarily occurs during changes in the operating conditions, such as start-up/shutdown (SU/SD), fuel supply fluctuations, and load variations.^11−13^ Especially for automotive applications, such transient conditions are inevitable because of starting and stopping of the vehicle.^4,14^ Pei and co-workers divided the working conditions in a PEMFC into four categories: load changing, SU/SD, high power, and idling. They found that the SU/SD alone contributes ∼33% to the degradation of the cathode, even though the fuel cell was only fully shut down once per hour of driving time.^11−13^ The reason for this disproportionate contribution of SU/SD is that uncontrolled transient voltage spikes up to 1.4 V_RHE_ occur during the SU/SD.^15^ These cause significant degradation in Pt because of the rapid oxidation/reduction and corrosion of the carbon support.^16^ Indeed, transient degradation of the Pt catalyst has been found to occur at a much faster rate compared to steady-state operation, where such high potentials are rarely reached.^12,18−22^ Son and co-workers verified this in their publications, where they found that the Pt loading at the cathode decreases by half just after 1500 SU/SD cycles at 100% relative humidity and noticed a significant decrease in the current density and ECSA.^9,23−25^ It has been shown that the reduction in ECSA is closely linked to the dissolution of Pt nanoparticles.^6,7,26^ In turn, Pt dissolution has been linked to oxidation/reduction of Pt,^27−29^ highlighting the key role of Pt oxides in fuel cell degradation.

A point of debate is the mechanism by which Pt oxide formation/reduction is linked to the degradation mechanisms that occur in fuel cells. Because Pt dissolution can occur during both oxide formation and reduction, as well as under (highly anodic) potentiostatic conditions, several types of dissolution reactions seem to occur. Oxidative, reductive and chemical dissolution reactions have all been proposed, each with subvarieties depending on whether Pt, Pt–OH_ads_, Pt–O_ads_, PtO, or PtO_2_ is involved.^27,28,30−35^

An important reason for the coexistence of this plethora of proposed Pt dissolution mechanisms is that the composition of the oxides involved in the process is not known. For equilibrium conditions, it has been established that electrochemical oxidation of Pt at potentials above ∼1 V_RHE_ yields an amorphous oxide layer, which typically contains a mixture of oxidation states.^20,34,36,37^ Even for the very early stages of oxidation, the entire oxidation state range of Pt^δ+^, Pt^2+^, and Pt^4+^ can be detected. The question now arises whether this also holds for the transient conditions relevant to the start-up or shutdown of fuel cells. In addition, the reduction potential for Pt oxides lies within the range 0.9–0.5 V_RHE_ and the operating potential of fuel cell is ∼0.7 V_RHE_,^12^ making it uncertain whether complete reduction will be achieved after a start-up pulse.

In this publication, we address these questions through the combination of in situ X-ray photoelectron spectroscopy (XPS), X-ray absorption spectroscopy (XAS), and electrochemical measurements. To enable the in situ measurements, we made use of our previously developed confined electrolyte approach,^20,38,39^ in which the Pt nanoparticles are sandwiched between a proton exchange membrane and a graphene window that separates the wet electrochemical environment from the vacuum of the spectroscopy setup. Using this methodology, we investigated the oxidation behavior of platinum during steady state and transient conditions. Detailed XP spectra show the evolution of the Pt surface structure during both oxidation and reduction. On the basis of this data, a model is derived to predict the oxidation charge measured in electrochemical measurements. We show that this model is in good agreement with the oxidation charge measured during SU/SD potential spikes reproduced in an electrochemical cell, thus underlining the universality of the model even for transient conditions.

## Methods

### Electrochemical Measurements

The electrochemical measurements were done in a homemade electrochemical cell. The cell utilizes a three-electrode setup, with a platinum wire (ChemPUR, 99.9%) as a counter electrode and an RHE as a reference electrode. Experiments were done in a two-compartment glass cell cleaned with acidic potassium permanganate (0.5 M H_2_SO_4_ solution (Sigma-Aldrich, 95.0–97.0%) containing 1 g L^–1^ KMnO_4_ (Sigma-Aldrich, ≥ 99.0%)) and dilute piranha solution (∼1 M H_2_SO_4_ (Sigma-Aldrich, 95.0–97.0%) and ∼6% H_2_O_2_ (Merck KGaA, 35%)) followed by repetitive boiling in ultrapure water ((Merck Milli-Q IQ 7000, <5 ppb total organic content (TOC), 18.2 MΩ cm at 298 K)). The working electrode was prepared by sputter depositing platinum onto a glassy carbon substrate. The catalyst loading was kept at 3–3.5 nm, as verified using a quartz crystal microbalance. Throughout this manuscript, the catalyst loading here is expressed as layer thickness. After Pt deposition, a thin layer of Nafion ionomer (Sigma-Aldrich, 5% solution) was deposited on the Pt/C electrode by drop casting. The Nafion drop on the working electrode was dried in a lab-made desiccator to give a uniform layer of ionomer on top of the platinum nanoparticles. The Nafion coated working electrode was transferred to the cell from the desiccator covered in a droplet of ultrapure water to avoid contamination from the ambient. The electrolyte used in the cell was prepared at a concentration of 0.1 M using a sulfuric acid stock solution (Sigma-Aldrich, 99.9% solution) and ultrapure deionized water (Merck). Before the start of the experiments, the Pt wire used as the counter electrode was flame-annealed and cooled in ultrapure water and the working electrode was cycled between 0.05 and 1.3 V_RHE_ to verify the quality and the cleanliness of the solution. The experiment was carried out using 0.1 M H_2_SO_4_. The setup was connected to a potentiostat (AutoLab, VSP-300) to conduct the electrochemical experiments. The maximum vertex potentials applied during the experiments were 0.05 V to 1.4 V_RHE_. All the experiments are carried out at room temperature using a static electrode configuration with a hanging meniscus maintained between the electrolyte and the working electrode.

### In Situ XPS

**Figure 1:**
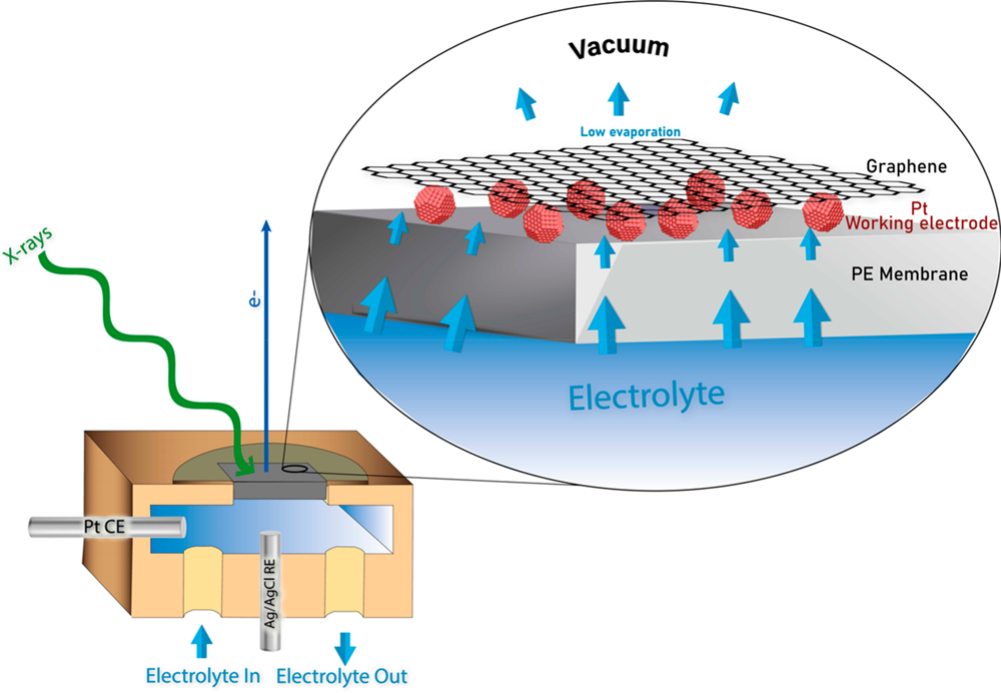
Schematic diagram and functioning of the in situ XPS cell.

For the current application, a special arrangement of an in situ electrochemical cell was used. The construction of this cell has been described in detail in one of our earlier publications^20,39,38^ so here only a brief overview is given. The cell is made up of platinum nanoparticles sandwiched between a proton exchange membrane and a graphene layer, as shown in Figure 1. A Pt wire (ChemPUR, 99.9% pure) is used as a counter electrode and a Ag/AgCl reference electrode, both housed in a flow channel behind the membrane. A detailed account of the sample preparation of the in situ spectroscopy cell is given in the Supporting Information.

For the purpose of analysis later, the potentials were converted to an RHE scale. The purpose of the graphene layer is to serve as an electrical contact between the catalyst nanoparticles even if they are scattered, as well as to prevent excessive evaporation of the electrolyte into the vacuum when the in situ cell is being examined by XPS and XAS. Because of a steady flow of the electrolyte diffusing through the proton exchange membrane combined with reduced evaporation because of the graphene capping, we can probe the catalyst under wet electrochemical conditions. It has been investigated previously that the graphene layer installed on top of the catalyst layer is transparent to photoelectrons (>300 eV kinetic energy (K.E.) for single-layer graphene and >400 eV K.E. for double layer graphene).^20,38^

For the purpose of this study, we use double-layer graphene as our electron transparent window. The platinum (Electronen-Optik-Service GmbH target) was deposited on the proton exchange membrane using a Cressington sputter coater, yielding a uniform layer thickness of approximately 2–3 nm.

The proton exchange membrane used in the cell was Nafion 117 (Ion Power), which allows the diffusion of water and protons to and from the Pt particles. For in situ XPS examination, the near-ambient pressure XPS end station (NAPXPS1) of the ISSS beamline at the BESSY II/HZB synchrotron facility in Berlin, Germany, was utilized. There were no gases introduced into the cell and the chamber pressure was determined at 0.05–0.15 mbar. Regarding the XAS measurements, a partial electron yield signal was collected by the analyzer at a kinetic energy of 385 eV. Prior to the measurements, the potential was cycled between the 0.05 and 1.3 V_RHE_ several times at 50 mV s^–1^ beforehand to prevent any memory effects from interfering with the results.

During the measurements, care was taken that the cell assembly did not endure beam damage. Therefore, all the individual measurements were taken at fresh spots on the surface.

The data from X-ray photoelectron spectroscopy experiments were processed in *CASA XPS* software. The platinum 4f spectra were deconvoluted into peaks and fitted using four doublets corresponding to Pt^0^, Pt^2+^, Pt^δ+^, and Pt^4+^ with corresponding binding energies taken from the literature^40^ and the spin–orbit splitting between the doublets kept constant at +3.33 eV. Details of the fitting procedure are given in the Supporting Information section 4.

## Results and Discussions

**Figure 2:**
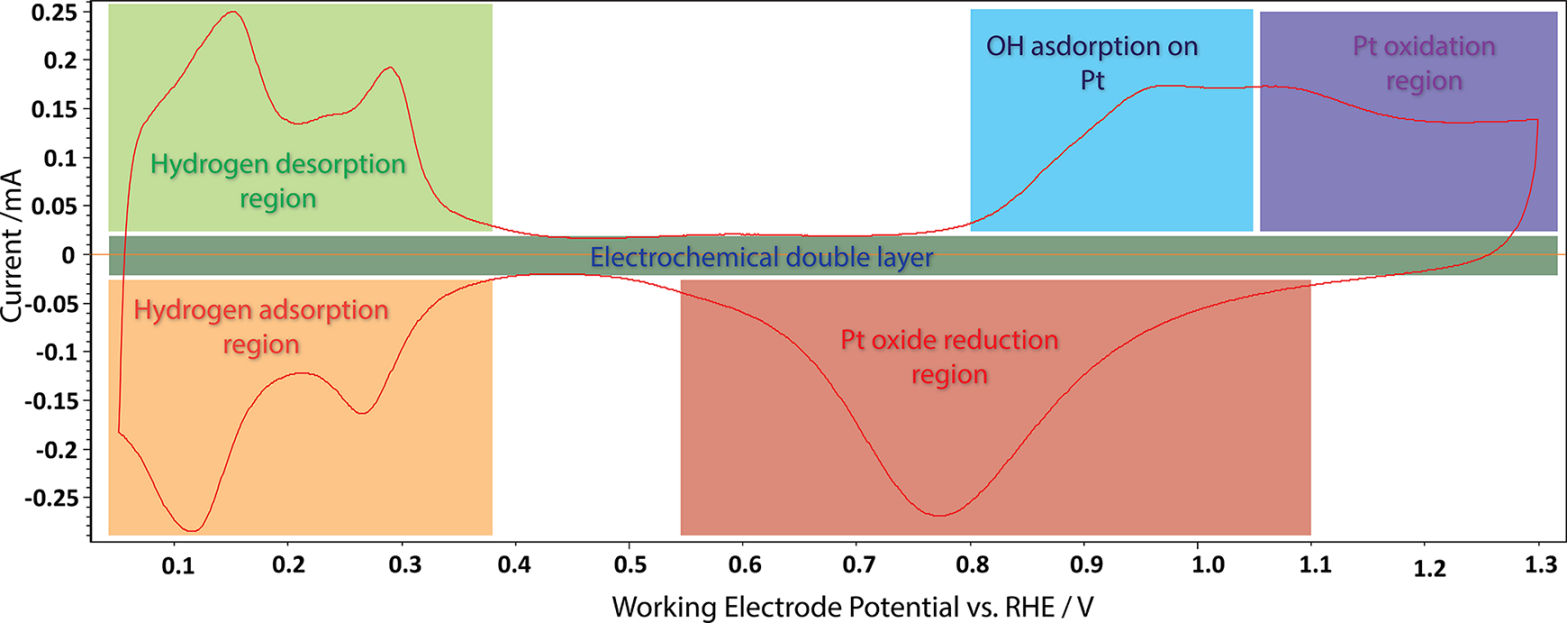
Cyclic voltammogram recorded from the electrochemical cell for Pt nanoparticles on a glassy carbon support with a Nafion film covering in an Ar-saturated 0.1 M H_2_SO_4_ electrolyte at a sweep rate of 50 mV s^–1^.

For our electrochemical characterization, accurate potential control of the working electrode is essential. We therefore employed a model catalyst that could be inserted into a classical three-electrode glass cell with an RHE reference electrode. As described in the Methods, our model catalyst contains the principal components of a fuel cell cathode, consisting of a glassy carbon support coated with Pt nanoparticles and covered with a thin layer of Nafion. As shown in Figure 2, our model system yields a cyclic voltammogram that exhibits the characteristic features of clean Pt nanoparticles, in good agreement with the literature results^41^ and PEMFC performance studies carried out under gas diffusion electrode conditions (using Pt/carbon + Nafion ink).^42−44^

Typical hydrogen adsorption/desorption features are observed between 0.05 and 0.4 V_RHE_.^45^ The electrochemically active surface area (ECSA) of Pt was calculated via the area of the hydrogen desorption peaks in the cyclic voltammograms using eq 1:
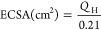
1Where *Q*_H_ represents the hydrogen desorption charge (mC cm^–2^) and 0.21 mC cm^–2^ stands for the charge that is needed to oxidize a monolayer of H adsorbed on Pt surface.^46,47^ The ECSA calculated using this expression will be used to normalize the oxidation/reduction currents in the following sections.

**Figure 3:**
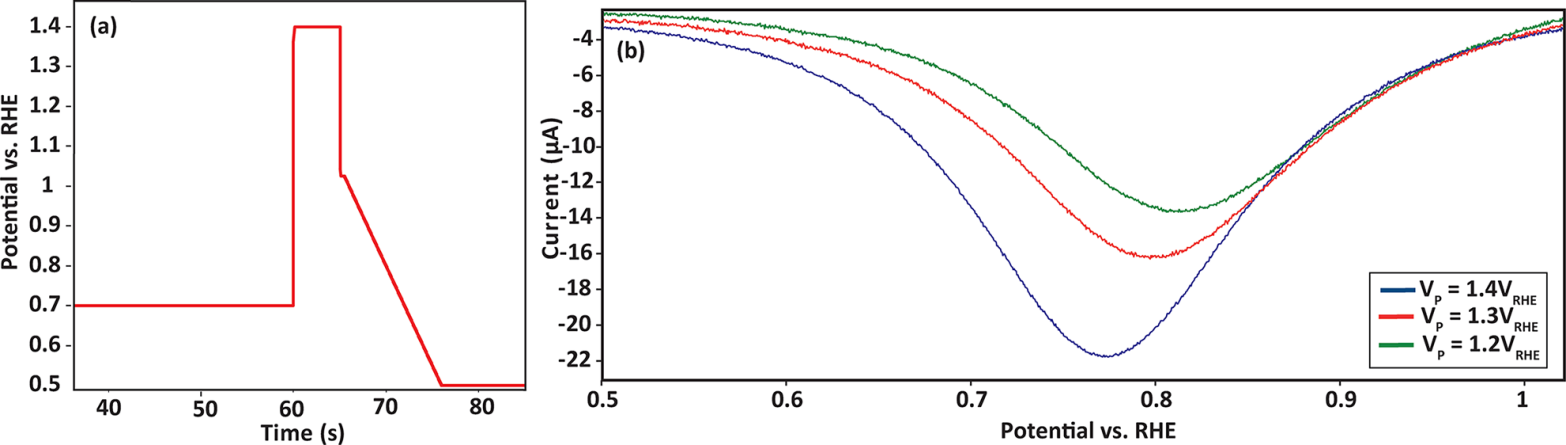
Potential transients to replicate and quantify oxide formation during fuel cell start-up and shut-down. (a) Voltage scheme applied. (b) Oxide reduction peak following pulses to various potentials (*V*_P_ = 1.2, 1.3, and 1.4 V_RHE_). Inset shows the typical current vs time response to the applied transient voltage scheme.

To replicate the potential spikes that occur during the start-up or shutdown of a fuel cell, we applied 5 s pulses to potentials in the range of 1.2 V_RHE_ to 1.4 V_RHE_. Modeling and local potential measurements during fuel cell start-up/shut-down^17,48,49^ showed that such high potentials indeed occur on fuel cell cathodes when the gas feed on the anode is changed from air to H_2_ or vice versa. The potential range between 1.2 V_RHE_ and 1.4 V_RHE_ is enough to oxidize the Pt particles. To determine the amount of oxide formed during the pulse, we reduced the oxide in a linear sweep voltammogram (LSV) at 50 mV s^–1^ (see Figure 3a).

The reduction peaks for different pulse potentials are shown in Figure 3b. It can be clearly observed that increasing the pulse potential results in a larger reduction peak, i.e., more oxide formation. Concomitantly, the reduction peak shifts to lower potentials, indicating that the oxidation/reduction reaction becomes increasingly irreversible for larger amounts of oxide, in line with the literature results.^50,51^

Using our in situ XPS approach, we explored the composition of the Pt oxides. To ensure that these measurements were conducted under representative wet electrochemical conditions, we recorded O K-edge XAS spectra during the experiments (see section S2). In line with previous work,^38,39^ the spectra show a clear fingerprint of liquid water, confirming that the catalyst layer was properly wetted. Having established this, we recorded XPS and XAS spectra while stepwise increasing and decreasing the potential.

**Figure 4:**
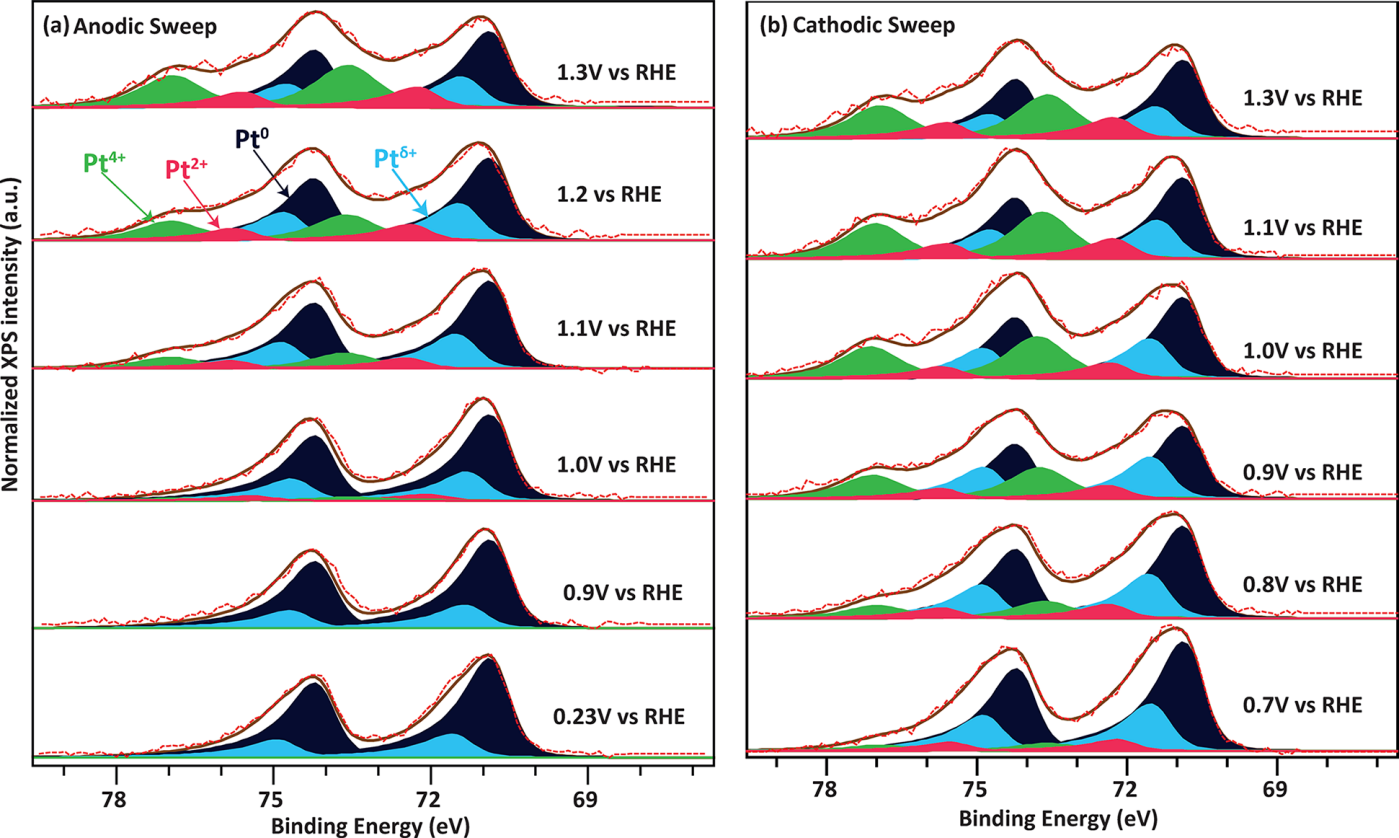
Pt 4f spectra of 2–4 nm Pt on Nafion during a stepwise increase and decrease in the potential. Excitation energy 600 eV, fit model: Pt^0^ (navy blue), Pt^δ+^ (sky blue), Pt^2+^ (pink), and Pt^4+^ (green). The dashed red line shows the raw XPS data and the brown line shows the curve fitting for the raw data. A Shirley background subtraction was applied to all spectra. (a) Stepwise anodization of the Pt nanoparticles. (b) Stepwise decrease of the potential. All Pt 4f spectra are normalized to the Pt^0^ peak at 71 eV.

Figure 4 shows the Pt 4f spectra recorded during the potential stepping. As described in the Methods, the collected spectra are fitted with doublets of Pt^0^, Pt^δ+^, Pt^2+^, and Pt^4+^. It can be observed that at low potentials (0.23 V_RHE_), the main contributor other than Pt^0^ is the Pt^δ+^ component. As highlighted in previous work, at potentials below the oxidation potential of Pt (∼0.9 V_RHE_), this contribution likely occurs because of the interaction of Pt surface atoms with adsorbates such as R-SO_3_^–^, OH, or O. These adsorbates carry a negative charge, inducing a δ+ charge in the Pt surface atoms. Above the Pt oxidation potential (1.0 V_RHE_), the formation of a Pt-PtO_*x*_ interface or 2D oxide could induce a similar effect on the Pt surface atoms, resulting in a similar Pt^δ+^ component. The intensity of the Pt^δ+^ component slightly increases as the potential is raised, particularly from 0.9 V_RHE_ to 1 V_RHE_, consistent with the increased coverage of O_ads_ and OH_ads_ in this potential range. The onset of the oxidation of Pt occurs at potentials higher than 0.9 V_RHE_, where we start to observe the presence of 2+ and 4+ oxidation states. Beyond 1.0 V_RHE_, a noticeable contribution of both Pt^2+^ and Pt^4+^ can be observed, indicating the onset of oxidation. These oxides are likely hydrous, but this could not be clearly resolved by the comparison of the in situ O K-edge data (Figure S4-a) to reference spectra of hydrous and nonhydrous PtO_2_. The appearance of both Pt^2+^ and Pt^4+^ at the onset of Pt oxidation indicates that a surface oxide with a complex structure is formed, in agreement with the poorly ordered structures observed by electrochemical scanning tunneling microscopy experiments^34^ and the broad Pt L_3_ absorption edge observed by Cuenya and co-workers.^37^ Hence, the surface oxidation of Pt clearly deviates from the regular Pt^0^ → Pt^2+^ → Pt^4+^ oxidation series predicted by Pourbaix diagrams.^53^

**Figure 5:**
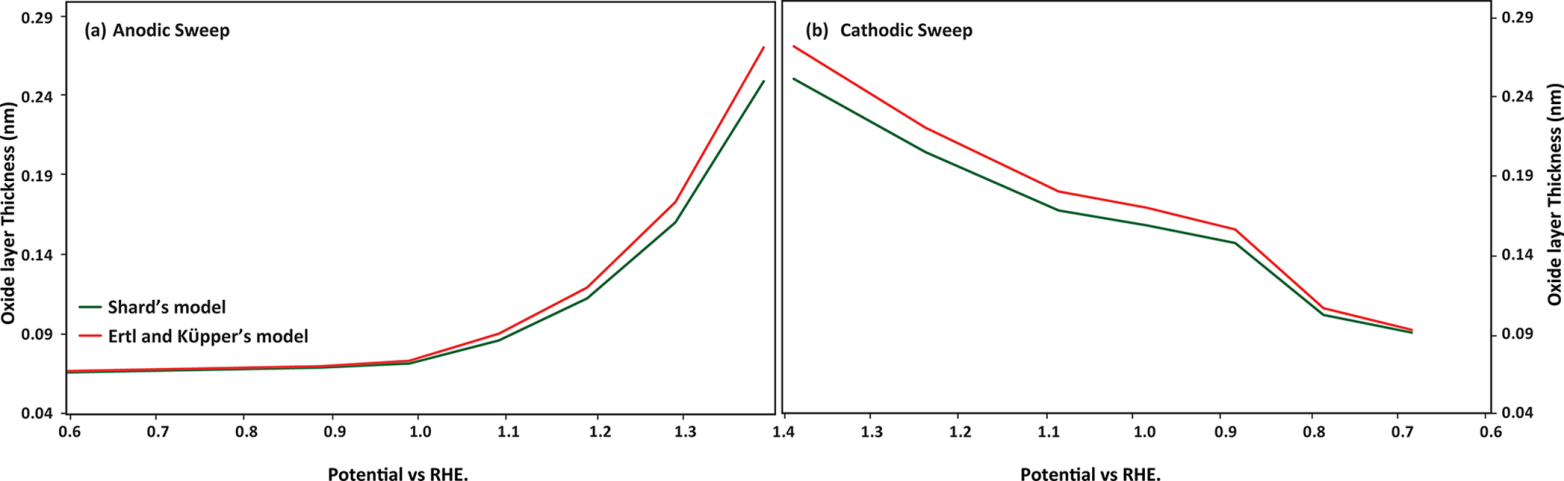
Modeling the oxide layer thickness by using Shard’s model (green) and Ertl and Kupper’s model (red) for (a) anodic sweep and (b) cathodic sweep.

Modeling of the observed oxide-to-metal ratio allows us to get a rough estimate of the thickness of the oxide layer. To obtain a reasonable estimate, it is important to consider the nanoparticle morphology of the sample, which boosts the XPS signal of the oxide shell relative to the metallic core. An exact consideration of the morphology is challenging, but we have made approximations using Shard’s model^54^ and Ertl and Küpper’s model^55^ (details in section S8). As shown in Figure 5, we obtain very modest oxide thicknesses, which do not exceed a monolayer. Hence, we conclude that only the outer Pt layer is oxidized in the potential range considered here. Note that the level of oxidation of the outer Pt layer likely varies for the different facets on the nanoparticles. However, this does not affect the quantitative analysis that we will discuss in the following paragraphs.

An essential difference between our XPS experiments and the transient pulses that occur during fuel cell start-up or shutdown is the time scale. For the XPS experiments, the potential is increased stepwise, at a rate of about 10 min per step. The pulses in a fuel cell, on the other hand, only last about 5 s.^17^ To test how representative the XPS data are of the oxidation processes under transient conditions, we used the surface structures observed in XPS to predict the electrochemical oxidation charge observed during the pulse experiments of Figure 3. The details of how the XPS data shown in Figure 4 were modified to represent the surface of the nanoparticle can be found in Section S5. For modeling the oxidation charge, we used the Pt ^δ+^/Pt^2+^/ Pt^4+^ ratio observed in Figure 4a, and the oxide thickness (∼1 monolayer). We assumed that a 1e^–^, 2e^–^, or 4e^–^ transfer was involved in creating Pt ^δ+^, Pt^2+^, and Pt^4+^, respectively. For example, this would lead to 2e^–^ transferred per surface Pt atom in order to form a 100% Pt^2+^ layer from a fully metallic surface, or 3e^–^ transferred for a 50% Pt^2+^, 50% Pt^4+^ layer. We also corrected for the contribution from surface adsorbed oxygen species by subtracting the number of electrons transferred at 0.9 V_RHE_ from the calculated total charge transfer. In this way, the oxidation charge per Pt surface atom from the XPS data was modeled for 1.2 V_RHE_, 1.3 V_RHE_, and 1.4 V_RHE_ (extrapolated). To obtain the oxidation charge from the electrochemical experiments, we integrated the reduction peaks in Figure 3b. We excluded the contribution of double layer capacitance as much as possible by subtracting the baseline from the LSV curve prior to integration. To express the oxidation charge in e^–^ per surface Pt atom, we normalized the measured charge using the H_UPD_ peaks obtained in cyclic voltammetry.

**Figure 6:**
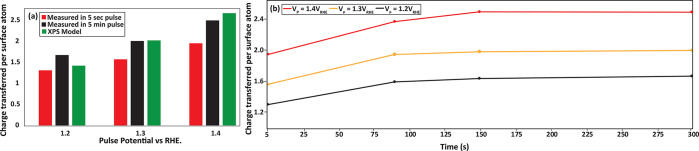
(a) Charge transferred during Pt oxidation, according to the measurements of Figure 3 and a model based on the XPS data. (b) Variation in charge transferred per Pt surface atom as a function the pulse duration.

Figure 6 displays the comparison between the XPS model and the measured oxidation charge transferred for 5 s and 5 min potential pulses. Clearly, the agreement is good, indicating that the XPS measurements have adequately captured the oxidation processes during the potential spikes. The agreement is particularly good for the 5 min pulses, which have a fairly similar time scale as the XPS measurements. However, even for the 5 s potential spikes, the XPS model overestimates the transferred charge by only ∼25%. The reason for this is that the time scale of the oxidation has only a modest impact on the formed oxide. This is clearly visible in pulse oxidation experiments with varying pulse duration (see Figure 6b). The vast majority of the Pt oxidation occurs with the first few seconds as shown in Figure 6b (see also Figure S5 for in situ XAS confirmation of this), whereas only some ∼25% additional oxidation charge is collected if the pulses are prolonged to several minutes. This indicates that the oxidation kinetics are fast, which is made possible by the fact that only the outer surface layer is oxidized in the potential range discussed here. Such surface oxidation does not require the (slow) diffusion of ions through the oxide layer, making it much faster than the bulk oxidation of Pt.^56^ As a result, we can conclude that our XPS data are representative of the oxides formed during transient potential spikes, i.e., that a surface layer of mixed oxidation state will also be formed under these conditions as well.

We will now shift our attention to the reduction of the oxides. Following the anodization up to 1.3 V_RHE_ in our XPS experiments, the potential was stepped down (cathodic sweep). Consistent with the typical irreversibility of Pt oxidation^26,31,57^ (see also Figure 2), the reduction of the Pt^2+^ and Pt^4+^ components follows a significantly different path than their formation (Figure 4 a, b and Section S2). Little change in the proportion of oxidation states is observed down to 1.0 V_RHE_. At lower potentials (Figure 4b), we see a sharp reduction in the amount of oxide, primarily in the Pt^4+^ component. It is important to note is that despite the long time that was allowed for the cathodic sweep (∼10 min per potential step), remnants of oxidized Pt remain on the surface of the particles at 0.9 V_RHE_ and 0.8 V_RHE_, and to some extend even at 0.7 V_RHE_. Because the typical operating range for fuel cell cathodes is about 0.7 V_RHE_ to 0.9 V_RHE_, this suggests that some oxides remain on the surface after a potential spike during start-up. Such oxide remnants would likely block active sites.^6,31,58^

**Figure 7:**
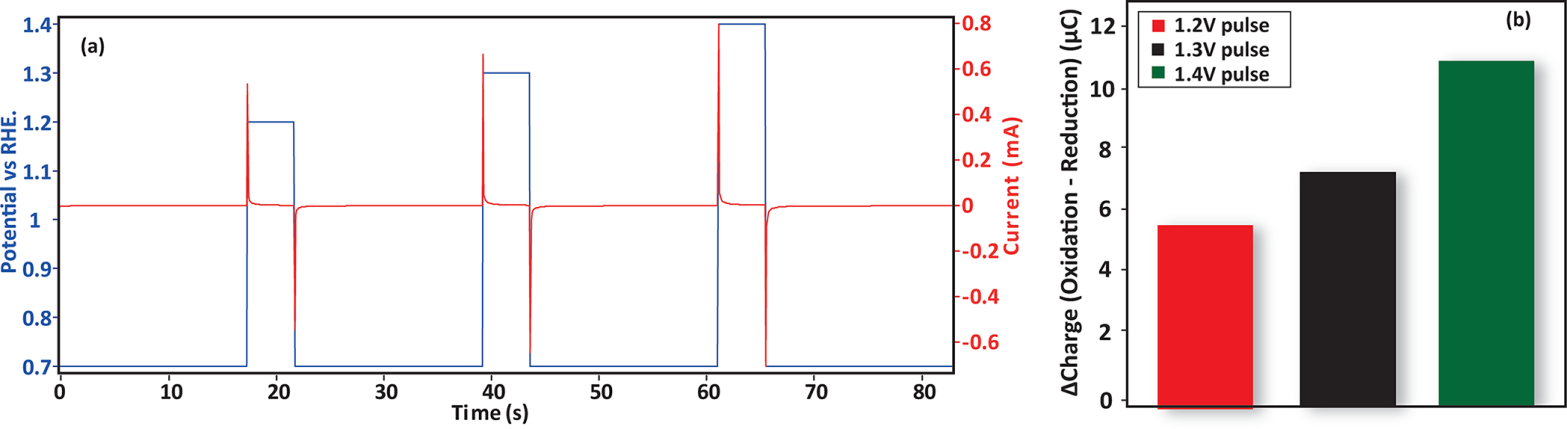
Consecutive pulse experiment: (a) applied voltage scheme and (b) difference between oxidation and reduction charge transfer.

To further visualize the remnants of oxide following fuel cell start-up conditions, we conducted a transient voltammetry experiment, with three pulses of increasing potential on a baseline of 0.7 V_RHE_ (see Figure 7a). As shown in Figure 7b, this experiment results in the accumulation of oxidative charge, again confirming that oxides remain following the pulses. The amount of oxide remnants scales with the pulse potential which can be estimated from the percentage of the oxidative charge that is not recovered during the reduction during the potential pulses (∼6–8% of the oxidation charge). This can be explained by the larger amount of oxide that is formed at higher potentials. A second contributing factor, as Figure 3 showed, is that the irreversibility of the oxidation/reduction increases for higher pulse potentials.

In the light of the observations above, we can say that following a potential pulse that occurs during the start-up, a fuel cell operating at a potential of 0.7–0.8 V_RHE_ is likely to have oxides present on the catalyst surface that may affect the performance. Hence, mitigating potential spikes during the start-up of a fuel cell may not only help to reduce Pt dissolution, but could also suppress site blocking by oxide remnants.

## Conclusions

In summary, we established a relationship between the equilibrium and transient oxidation behavior of Pt by combining in situ spectroscopy and electrochemical measurements under fuel-cell-relevant conditions. We find that an oxide monolayer containing a mixture of Pt^δ+^/Pt^2+^/Pt^4+^ oxidation states is formed over a wide potential range under both equilibrium and transient conditions, contrary to the stepwise oxidation series predicted by the Pourbaix diagram. Oxidation during 5 s potential pulses, such as those occurring during the start-up and shutdown of fuel cells, results in an oxidation charge of only a ∼ 25% less than for an equilibrated surface. This indicates that even under such transient conditions, the surface oxide structure approaches equilibrium. Further investigation of the transient pulses revealed that the oxides formed during the pulse are not fully reduced at the typical fuel cell operating potential of ∼0.7 V_RHE_. This shows that a potential pulse during fuel cell start-up will leave leftover oxides that potentially block some of the active sites during operation. In a more general view, our findings serve as a starting point for obtaining a mechanistic understanding of the transient phenomena that govern fuel cell degradation during start-up and shutdown.

## Supporting Information Available

Details on changing Pt oxidation states with changing step potentials, details on catalyst wetting observed via in situ XAS, XPS data fitting parameters via *CasaXPS* software, details on data model used to correlate in situ XPS and electrochemical data, consecutive transient pulses observed via in situ XPS, prolonged pulses observed via in situ XAS, details of data models used to model oxide layer thickness on the nanoparticle (PDF)
