# Supplementary material for: Structural Model for Transient Pt Oxidation during Fuel Cell Start-up Using Electrochemical X-ray Photoelectron Spectroscopy
Source: ACS Appl Mater Interfaces. 2022 Jul 29;14(31):36238–45. doi: 10.1021/acsami.2c09249 (PMC9376923; doi:10.1021/acsami.2c09249)
Supplement: Supplementary file 1 — am2c09249_si_001.pdf [file am2c09249_si_001.pdf]

Supporting Information

A structural model for transient Pt oxidation during fuel cell start-up using electrochemical X-ray photoelectron spectroscopy

Hassan Javed[1*], Axel Knop-Gericke[2,3], Rik V. Mom[1]

[1]Leiden Institute of Chemistry, Leiden University, PO Box 9502, 2300 RA Leiden, The Netherlands
[2]Fritz Haber Institute of the Max Planck Society, Faradayweg 4-6, 14195 Berlin, Germany
[3]Max-Planck-Institute for Chemical Energy Conversion, Stiftstrasse 34-36, 45413 Mülheim an der Ruhr, Germany

*h.j.nagra@lic.leidenuniv.nl

Supporting Information (SI)

1. *In situ* cell sample preparation

The Polymer electrolyte membrane onto which Pt nanoparticles are to be deposited, is Nafion 117 supplied by Sigma-Aldrich. The membrane supplied by the vendor was cut into round discs of 11 mm in diameter, followed by the process of activation and cleaning. The Nafion membrane needs to be cleaned of the carbonaceous impurities. For this purpose, the Nafion discs were treated with a 3% H_2O_2 solution at 80°C for 2hr and then with dilute H_2SO_4 solution (0.5M) for the same duration and temperature.

The membrane electrode assembly (MEA) is prepared by sputter depositing Pt nanoparticles onto a polymer exchange membrane using a Pt target (ChemPUR GmbH, 99.5% purity) using a DC magnetron sputter coater 208HR by Cressington (Watford, UK). The process was carried out in an Argon atmosphere at 0.1 mbar and a pre-programmed sputtering current of 40 mA. The thickness of the Pt layer was controlled by automated MTM-20 high resolution thickness controller which constantly monitors the thickness of the deposited film as a function of the programmed density (for Pt = 19.45 g.cm^{-3}) of the material of interest and the particle deposition was calibrated using TEM. For our study, the thickness of the Pt layer was controlled between 3-4 nm.

Following the preparation of the MEA, a graphene layer is deposited on top of the Pt nanoparticles to impede the escape of the electrolyte during the spectroscopic measurements as well as to serve as an X-ray/photoelectron transparent window (>300 eV) and as the electrical contact with the Pt nanoparticles. Graphene is deposited using a wet chemical method. Graphene supported on copper (Graphenea SA) was etched in a 40 g/L solution of ammonium sulfate overnight, dissolving copper and leaving the graphene layer floating on the liquid surface which is visible against a white background. The solution was then exchanged with pure water and the MEA was placed inside the water below the graphene and the liquid level was the lowered such that the graphene layer would land on the membrane. The prepared sample with graphene was dried at room temperature and proper placement of graphene was ensured on the membrane by visual inspection.

2. Change in concentration of different oxidation states of Pt with changing potential

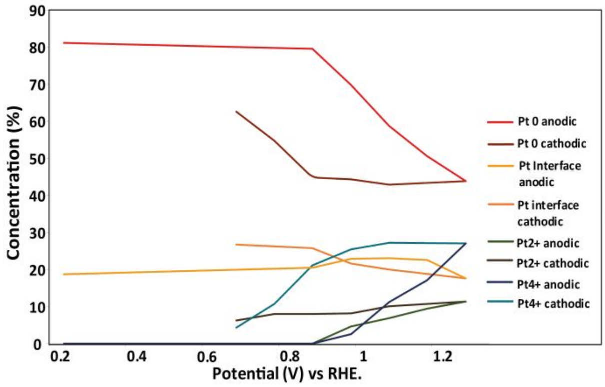

	Potential vs. RHE	Pt 0	Pt Interface	Pt 2+	Pt 4+
Anodic	0.238241	81.18	18.74	0.07	0
	0.888241	79.5	20.5	0	0
	0.988241	69.82	22.91	4.69	2.58
	1.088241	58.77	23.04	6.96	11.24
	1.188241	50.57	22.63	9.54	17.26
	1.288241	43.89	17.6	11.41	27.09
	1.388241	36.42	12.57	13.28	37.73
Cathodic	1.088241	42.92	20.05	10.22	27.25
	0.988241	44.45	21.73	8.23	25.59
	0.888241	44.91	25.77	8.09	21.23
	0.788241	54.83	26.29	8.04	10.83
	0.688241	62.65	26.74	6.25	4.34

Figure S 1: Percentage concentration of Pt oxidation states vs applied potential

Figure S 1**Error! Reference source not found.** shows a detailed picture of Pt oxidation observed at different step potentials. The difference in compositions at, for example, 0.9V_{RHE} and 1.0V_{RHE} during the cathodic and the anodic parts show the electrochemical irreversibility at which the oxide species on platinum are formed and reduced.

3. Catalyst wetting observed via XAS spectrum

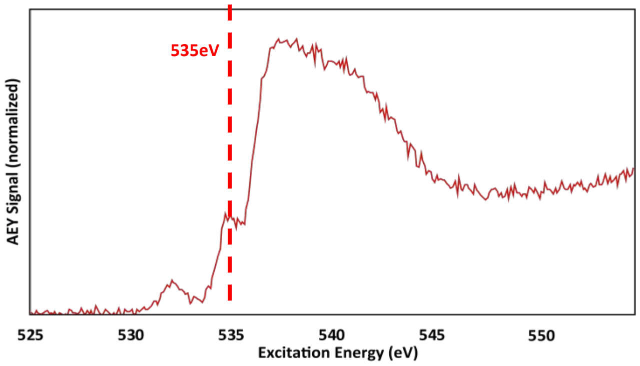

Figure S 2: Effect of graphene covering on O-K edge spectra recorded at 0.7V_{RHE}

One of the most important features of the *in situ* spectroscopy cell is its ability to ensure the wettability of the catalyst under vacuum conditions. To check the wetting of the catalyst, we use in situ O K-edge XAS spectroscopy, which is conducted during the in situ XPS experiments in the same geometry. One of the most important features in the O K-edge spectrum that we have used to track the wetting of the Pt nanoparticles is the peak at 535 eV, which is very typical for liquid water[1,2]. The O K edge spectrum shown in Figure S 2 is measured at a low potential of 0.7V_{RHE} to avoid the contribution from platinum oxides. To reduce the noise in the PEY spectrum, an average of several spectra was taken at several locations on the catalyst surface to avoid the effects of beam damage.

As shown in Figure S2, the 535 eV peak is very pronounced. This can only be explained by either the presence of liquid water, or by a significant contribution from graphene oxide to the spectrum. However, graphene oxide also produces a strong resonance at about 531.9 eV. In our case, only a weak contribution is visible, ruling out the possibility that functional groups on the graphene window have contributed significantly to the 535 eV peak[1]. Hence, we can say with certainty that the peak at 535eV in the O-K edge spectrum predominantly originates from water, i.e. that the catalyst layer was properly wetted during the in situ XPS experiments.

4. **XPS Data fitting parameters**

The raw XPS data was processed using CasaXPS Version 2.3.23. A Shirly background subtraction was used for all the datasets. The Lorentzian LF line shape was employed, which is an extension of Lorentzian LA line shape, the purpose of which is to limit the intensity of the asymmetric tails. It fits the data on the basis of 4 parameters;α, β, w and m. Varying 'α' and 'β' results in increasing or decreasing the spread of the tail for the Lorentzian curve, thereby affecting how steep are the edges of the line shape. 'm' is the integer specifying the Lorentzian convolution by the gaussian function and 'w' is the dampening factor to force the tails of the curve to reduce towards the limits of integration. An asymmetric form of Lorentzian LF line shape function was used for fitting the raw Pt 4f spectra. It is known through literature that, as a consequence of spin-orbit coupling, the electrons that leave Pt 4f orbital, generate two distinct energy peaks in the XPS spectrum categorized as $4f_{5/2}$ and $4f_{7/2}$. Hence doublets for Pt^0 $Pt^{\delta+}$, Pt^{2+} and Pt^{4+} were used, with a constant spin-orbit splitting of 3.34eV and a peak area ratio of 3:4 for the $4f_{5/2}$ and $4f_{7/2}$ peaks. The details of the fitting parameters are shown in the table below.

	Pt^0	$Pt^{\delta+}$	Pt^{2+}	Pt^{4+}
**Line shape**	LF(0.63,2,20,70)	LF(0.63,2,20,70)	LF(0.63,2,20,70)	LF(1.8,2,10,0)
**Peak Position**	70.81 - 70.7999	72 - 71.2	72.7 - 72	Determined at highest potential of the dataset
**FWHM**	Free	Same as Pt0	Same as Pt0	Determined at highest potential of the dataset

Figure S 3: Fitting parameters for Pt 4f spectral decomposition

The ranges for the peak positions of different Pt oxidation states were determined in accordance with the literature, especially cited in detail by Savelena and their group[3] and previous work [1]. In order to accommodate the variations in the configuration of the beam energy for different experiments, a slight variation in the binding energy for Pt^0 (± 0.1eV) was permitted. The line shape parameters were determined in similar fashion as in previous work [1]. The Pt^{4+} line shape, peak position, and FWHM were determined by free fitting of a highly oxidized 4nm Pt sample produced at $1.85V_{RHE}$, which displays a well-resolved Pt^{4+} peak. The line shape and FWHM of the other components was determined at low potential, where the metallic contribution is dominant. Note, however, that there is no potential at which only one component could be fitted. This is in line with the notion that the Pt surface atom are always in contact with adsorbates (e.g. H_2O, OH, O, R-SO_3^-), which generates a $Pt^{\delta+}$ and a bulk Pt^0 peak. The consistency of the fit model was ensured by applying it to several data sets.

5. **Charge transfer calculation for XPS data**

As mentioned in the main text, an oxidation charge transfer value was calculated based on the ratio of oxidation states observed in the XPS data. Since the comparison of this value is to be drawn with the measured surface oxidation charge of Pt nanoparticles in the electrochemical cell, the XPS data (as shown in Figure Figure S 1) had to be corrected to represent the surface of the nanoparticle only. To estimate the fraction of the total XPS signal emerging from the nanoparticle surface, it was assumed that at $1.4V_{RHE}$ the surface layer is completely oxidized (as confirmed by oxide layer

thickness modelling). Hence, the signal from Pt^0 at $1.4V_{RHE}$ arises from subsurface while $Pt^{\delta+}$, Pt^{2+} and Pt^{4+} species constitute the surface signal, which comes out to be ~63% of the measured XPS signal. The intensities of the $Pt^{\delta+}$, Pt^{2+} and Pt^{4+} contributions shown in Figure Figure S 1 were rescaled using this number, so that they represent the fraction of the surface that was occupied by these species.

The basis of oxidation charge calculation is the formation of a monolayer of oxides on the Pt surface and considering the formation of $PtO(Pt^{2+})$, $PtO_2(Pt^{4+})$ and $Pt_{Interface\ oxide}(Pt^{\delta+})$ as $2e^-$, $4e^-$ and $1e^-$ transfer processes, respectively. The average no. of e- transferred by the surface Pt atoms was calculated for each value of the step potentials shown in Figure Figure S 1 as follows:

$$Avg.\,e-transferred = \frac{(x_{Pt\,\delta+} * 1) + (x_{Pt\,2+} * 2) + (x_{Pt\,4+} * 4)}{0.63}$$

(2)

Where $x_{Pt\,i}$ is the measured fraction of Pt species and 0.63 represents the fraction of the XPS signal originating from the surface of the nanoparticle. The calculated average number of electrons transferred has to be corrected to exclude the contribution from adsorbed oxygen or OH species (O_{ads}/OH_{ads}) that are already present at the base potential of the pulses in the electrochemical experiment (0.7 V_{RHE}). We approximated this correction by subtracting the number of electrons transferred at $0.9V_{RHE}$ from the ones calculated at higher potentials:

$$Corrected\ e-transferred\ at\ V_{pulse} = (Calculated\ e-transferred\ at\ V_{pulse}) \\ -(e-transfred\ at\ 0.9V_{RHE}) \qquad (3)$$

Where $V_{pulse} = 1.2, 1.3\ and\ 1.4V_{RHE}$.

6. XAS experiments with consecutive voltage pulses

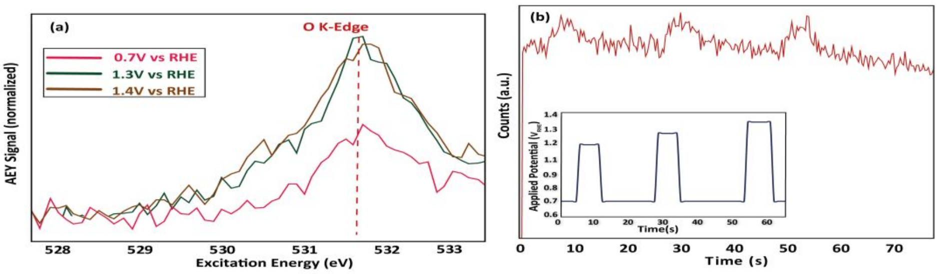

Figure S 4: XAS spectra for applied pulse potentials. (a) Inset shows the consecutive pulses applied and the main set shows tracked XAS spectrum peak at 531.9eV while (b) shows the same peak at different pulse potential in overlay arrangement

Figure Figure S 4(a) shows XAS spectra tracking the O K-edge peaks at 531.9eV, which is associated with the formation of Pt oxides[1] exhibiting significant oxidation at higher anodic potentials. It is also indicated in the literature that a high O K-edge intensity between 529.5eV and 532eV at potentials as high as $1.3V_{RHE}$ and $1.4V_{RHE}$ can be associated with the formation of a considerable amount of PtO_2[4–6] which is consistent with our XPS results(see section Figure S 1). The O K-edge spectrum in the main set of Figure Figure S 4(b) show a corresponding sharp spike at 531.9eV for every potential pulse applied as shown in the inset, indicating significant oxidation during SU/SD pulses. It is also

noticeable that for each of these pulse responses, the rise is much steeper than the fall which tells us about the kinetics of oxidation (rise) being much faster than reduction (fall).

7. Extended potential pulse experiment (XAS)

To observe the oxidation behavior for prolonged oxide conditioning times, the applied potential was raised instantaneously from $0.7V_{RHE}$ and to $1.4V_{RHE}$ and maintained, with the intensity of the O-K edge at 531.9eV tracked as a function of time. It can be seen clearly from Figure S 5 that the O-K edge peak shows a plateau following an instantaneous spike in counts as a function of potential pulse, indicating swift surface oxidation of Pt nanoparticles after which the role of diffusion becomes more important and oxidation slows down. This observation confirms the conclusion that was drawn from similar electrochemical experiment indicated in **Error! Reference source not found.**.

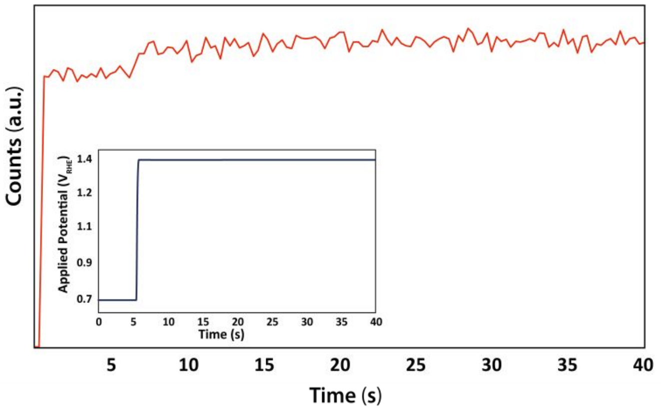

Figure S 5:Time resolved O-K edge peak intensity at 531.9eV (main set) for potential hold scheme at $1.4V_{RHE}$ (inset)

8. Modelling of oxide layer thickness

The initial particle size before oxidation was taken as 2.5nm. All the components highlighted in the XPS data fitting (see **Error! Reference source not found.**) were incorporated in the thickness modelling for both the models discussed below.

a) Shard's model

Shard's model [7] provides us with a straightforward approach to estimate the thickness of the overlayer on a nanoparticle as a function of the normalized XPS intensities observed experimentally.. In our paper, the fundamental equation adopted from the Shard's model to estimate the oxide layer thickness is as follows;

$$T_{NP} = \frac{T_{R\sim 1} + \beta T_o}{1+\beta} \tag{4}$$

Where T_{NP} is the thickness of the oxide layer, while $T_{R\sim 1}$, β and T_o are variables which are calculated as a function of α, β and core radius of the nanoparticle (*R*). *α* and β are calculated as follows:

$$\alpha = \frac{1.8}{A^{0.1}B^{0.5}C^{0.4}} \tag{5}$$

$$\beta = \frac{0.13\alpha^{2.5}}{R^{1.5}} \tag{6}$$

Based on previous TEM data[1], we estimate the core radius to be around 2.5 nm for unoxidized particles.

Shard's model in essence takes into account one[1] component in the core of the particle and one component in the oxide shell. For our model, a weighted average of the relative electron attenuation length and normalized XPS intensities had to be incorporated into variables A and C to take into account all the oxide components identified in the XPS analysis:

$$A = \left(\left(\frac{x_{Pt\,\delta+}}{x_{Pt\,\delta+} + x_{Pt\,2+} + x_{Pt\,4+}}\right)\left(\frac{I_{Pt\,\delta+}}{I_{Pt}}\right)\left(\frac{I^{o}_{Pt}}{I^{o}_{Pt\,\delta+}}\right)\right) + \left(\left(\frac{x_{Pt\,2+}}{x_{Pt\,\delta+} + x_{Pt\,2+} + x_{Pt\,4+}}\right)\left(\frac{I_{Pt\,2+}}{I_{Pt}}\right)\left(\frac{I^{o}_{Pt}}{I^{o}_{Pt\,2+}}\right)\right) + \left(\left(\frac{x_{Pt\,4+}}{x_{Pt\,\delta+} + x_{Pt\,2+} + x_{Pt\,4+}}\right)\left(\frac{I_{Pt\,4+}}{I_{Pt}}\right)\left(\frac{I^{o}_{Pt}}{I^{o}_{Pt\,4+}}\right)\right) \quad (7)$$

$$C = \left(\left(\frac{x_{Pt\,\delta+}}{x_{Pt\,\delta+} + x_{Pt\,2+} + x_{Pt\,4+}}\right)\left(\frac{L_{Pt\,\delta+}}{L_{Pt}}\right)\right) + \left(\left(\frac{x_{Pt\,2+}}{x_{Pt\,\delta+} + x_{Pt\,2+} + x_{Pt\,4+}}\right)\left(\frac{L_{Pt\,2+}}{L_{\,Pt}}\right)\right) + \left(\left(\frac{x_{Pt\,4+}}{x_{Pt\,\delta+} + x_{Pt\,2+} + x_{Pt\,4+}}\right)\left(\frac{L_{Pt\,4+}}{L_{Pt}}\right)\right) \quad (8)$$

where

$x_{Pt\,i}$ (where $i= \delta+, 2+, 4+$) : concentration of $Pt^{\delta+}$, Pt^{2+} and Pt^{4+}

$\frac{I_{Pt\,i}}{I_{Pt}}$ = ratio of measured XPS intensity of oxide component i in the shell and metallic Pt (assumed to be) in the core

$\frac{L_{Pt\,i}}{L_{Pt}}$ = ratio of the attenuation length of the photoelectrons arising from material i when travelling in the shell to the attenuation length of the photoelectrons arising from material i when traveling in the core

$\frac{I^{o}_{Pt}}{I^{o}_{Pt\,i}}$ = ratio of XPS intensities for a flat, pure Pt metal surface and a flat pure version of material i.

B is the ratio of attenuation lengths of the core and shell material for photoelectrons traveling in the shell. Since the kinetic energy of the photoelectrons of all species discussed here is roughly the same, B can be taken as 1.

The pure component intensities are estimated in this model using the following expression;

$$\frac{I^{o}_{Pt}}{I^{o}_{Pt\,i}} = \frac{N^{o}_{Pt}.\,L_{PtinPt}.G(E_{Pt})}{N^{o}_{Pt\,i}.\,L_{\,i\,in\,i}.\,G(E_{Pt\,i})}$$

(9)

In the above equation, N^{o}_{Pt} and $N^{o}_{Pt\,i}$ are the number densities (g.cm^{-3}) of pure metal and pure oxide components (Pt= 21.45, $Pt^{\delta+}$($Pt_{\text{interface oxides}}$)= 20, Pt^{2+}(PtO)= 14.9, Pt^{4+}(PtO_2)= 10.2), L_{PtinPt} is the attenuation length in pure metallic Pt, $L_{\,i\,in\,i}$ is the attenuation length in material i, and $G(E_{Pt})$and $G(E_{Pt\,i})$ are the spectrometer transmission factors as a function of photoelectron kinetic energies. Since the kinetic energy of the photoelectrons of all species discussed here is roughly the same, the transmission factors are taken as equal and cancel out.

Note that the shell thickness T and the particle radius R are expressed in units of $L_{Pt\,i}$ in the model.

Ertl and Küpper's model

Ertl and Küpper's model is an alternative way to estimate the oxide layer thickness (d) on the nanoparticles[8]. The equation used in the modelling of the thickness is as follows;

$$d = L_{avg}.cos\theta.ln\left(1 + \frac{I_{ox}.I_M^o}{I_M.\,I_{ox}^o}\right) \quad (10)$$

Where L_{avg} stands for the average photoelectron attenuation length based on the weighted average of the metal and oxide species (done similarly as in Shard's model), θ is the take-off angle normal to the surface (taken as 57° here). The value of take-off angle is derived from the publication of Castner and coworkers, who have used similar values for nanoparticles of a comparable size as our application[9].

References

(1) Mom, R.; Frevel, L.; Velasco-Vélez, J. J.; Plodinec, M.; Knop-Gericke, A.; Schlögl, R. The Oxidation of Platinum under Wet Conditions Observed by Electrochemical X-Ray Photoelectron Spectroscopy. *J. Am. Chem. Soc.* **2019**, *141* (16), 6537–6544. https://doi.org/10.1021/jacs.8b12284.

(2) Merte, L. R.; Behafarid, F.; Miller, D. J.; Friebel, D.; Cho, S.; Mbuga, F.; Sokaras, D.; Alonso-mori, R.; Weng, T. C.; Nordlund, D.; Nilsson, A.; Cuenya, B. R.; Roldan Cuenya, B. Electrochemical Oxidation of Size-Selected Pt Nanoparticles Studied Using in Situ High-Energy-Resolution X - Ray Absorption Spectroscopy. *ACS Catal.* **2012**, *2* (11), 2371–2376. https://doi.org/10.1021/cs300494f.

(3) Saveleva, V. A.; Papaefthimiou, V.; Daletou, M. K.; Doh, W. H.; Diebold, M.; Zafeiratos, S.; Savinova, E. R.; Ulhaq-bouillet, C.; Diebold, M.; Zafeiratos, S.; Savinova, E. R. Operando Near Ambient Pressure XPS (NAP-XPS) Study of the Pt Electrochemical Oxidation in H 2 O and H 2 O/O 2 Ambients. *J. Phys. Chem. C* **2016**, *120* (1), 15930–15940. https://doi.org/10.1021/acs.jpcc.5b12410.

(4) Kaya, S.; Casalongue, H. S.; Friebel, D.; Anniyev, T.; Miller, D. J. Oxidation of Pt (111) under Near-Ambient Conditions. **2011**, *195502* (November), 1–5. https://doi.org/10.1103/PhysRevLett.107.195502.

(5) Costa, D.; Dintzer, T.; Arrigo, R.; Knop-gericke, D. A. Chemical Science In Situ Investigation of Dissociation and Migration Phenomena at the Pt / Electrolyte Interface of An. **2015**, 5635–5642. https://doi.org/10.1039/C5SC01421B.

(6) H.Yoshida, S. Nonoyama, Y. Yazawa, T. H. Quantitative Determination of Platinum Oxidation State by XANES Analysis. *Phys. Scr. T* **2005**, *115* (1), 813–815. https://doi.org/https://doi.org/10.1238/Physica.Topical.115a00813.

(7) Shard, A. G. A Straightforward Method for Interpreting XPS Data from Core-Shell Nanoparticles. *J. Phys. Chem. C* **2012**, *116* (31), 16806–16813. https://doi.org/10.1021/jp305267d.

(8) Ertl, G., Küppers, J., & Grasserbauer, M. Low Energy Electrons and Surface Chemistry. *Anal. Chim. Acta* **1987**, *199* (1), 272–273. https://doi.org/https://doi.org/10.1016/s0003-2670(00)82831-7.

(9) Techane, S. D.; Gamble, L. J.; Castner, D. G. Multitechnique Characterization of Self-Assembled Carboxylic Acid-Terminated Alkanethiol Monolayers on Nanoparticle and Flat Gold Surfaces. **2011**, 9432–9441.
